# Postpartum ovulation and early pregnancy in the menstruating spiny mouse, *Acomys cahirinus*

**DOI:** 10.1038/s41598-021-84361-z

**Published:** 2021-03-05

**Authors:** Jarrod McKenna, Nadia Bellofiore, Evdokia Dimitriadis, Peter Temple-Smith

**Affiliations:** 1 Department of Obstetrics and Gynaecology, Education Program in Reproduction and Development, Monash University, Melbourne, Australia; 2 The Ritchie Centre, Hudson Institute of Medical Research, Melbourne, Australia; 3 Department of Obstetrics and Gynaecology, The University of Melbourne, Melbourne, Australia; 4 The Royal Women’s Hospital, Melbourne, Australia

**Keywords:** Cell biology, Developmental biology, Medical research

## Abstract

Egyptian spiny mice are the only known species to have human-like menstruation and a postpartum ovulation. Unfortunately, no endocrine or morphological evidence has been provided for a postpartum ovulation in spiny mice, and while later stages of pregnancy have been well studied, early events including embryo implantation and spiral artery remodelling have not been reported. This study compared the sex steroid endocrinology and reproductive tract morphology of dams at eight timepoints (n = 40) postpartum to determine the timing of ovulation and the timing and invasiveness of embryo implantation in *A. cahirinus*. Reproductive tracts were fixed and stained for histology and immunohistochemistry, and plasma was prepared for enzyme-linked immunosorbent assay. Ovarian histology and estradiol-17B concentrations indicate ovulation within 48 h of parturition and then immediate resumption of follicular growth. Uterine histology and immunohistochemistry revealed progressive epithelial repair, endometrial growth and spiral artery assembly and remodelling in dams postpartum. Blastocysts were seen in the uterine lumen at day 4–5 postpartum and embryos had implanted superficially with minimal stromal invasion by day 5–6. This study provides further evidence for the unique, humanesque reproductive biology of spiny mice and for a postpartum ovulation using endocrine and morphological changes observed during early pregnancy. Taken together, our data suggest that spiny mice may act as appropriate models of human pregnancy disorders such as implantation failure or pre-eclampsia.

## Introduction

Several species of mammals experience a postpartum ovulation (PPO) during which they ovulate and copulate within 24 h of parturition^1^. In rodents, the PPO is primarily controlled by endogenous steroid hormones^2^ and maximises reproductive efficiency through a shorter generation interval between litters. In menstruating species, ovulation occurs spontaneously in response to a surge of estradiol and luteinizing hormone (LH)^3^. Following ovulation, formation of the corpus luteum (CL) and increased progesterone secretion induce terminal differentiation of endometrial stomal cells (spontaneous decidualisation; SD) in preparation for embryo implantation^4^. While these processes occur naturally during the primate menstrual cycle, ovulation, SD and embryo implantation have not been reported in any menstruating species postpartum; this is due to the suppression of ovarian function by lactational amenorrhea^5^.

Further, Gray, et al.^6^ reported anovulation for 45 days on average postpartum in non-breastfeeding women, compared to an average 189 days in those who consistently breastfed. In contrast, the Egyptian spiny mouse (Acomys *cahirinus*), the only rodent to have a primate-like menstrual cycle^7^, also experience a PPO^8^. Considering the highly consistent 40-day inter-birth interval^9^, PPO has been assumed to occur 24 h after litter delivery and the sequence has been used to estimate gestational and fetal age in recent studies^10–12^ owing to the lack of a discernible vaginal plug after mating^13^. Additionally, because spiny mice mothers actively nurse their young from birth (observations from our colony), and assuming spiny mice have similar endocrine control of ovulation as humans, dams would be expected to remain anovulatory whilst nursing.

Moreover, while murid rodents form the same type of placenta as humans (haemochorial), embryo implantation in mice is superficial with minimal trophoblast invasion compared to the interstitial, aggressive implantation in humans^4^. While spiny mice also form a haemochorial placenta^12^, the mode and receptivity to embryo implantation, and the endocrinology and morphology of the postpartum reproductive tract have not been characterised. Moreover, while spiral artery assemblies have been demonstrated in this species^14^, their subsequent remodelling during early pregnancy has yet to be confirmed. These basic observations are required to further assess the role of *A. cahirinus* as a suitable model for female reproductive function, and to understand the evolutionary biology of menstruation in this species. Therefore, this study aims to (1) identify the timing of PPO through evaluation of sex steroid endocrinology, (2) evaluate the gross morphology of the postpartum reproductive tract, and (3) to describe the timing, invasiveness and receptivity to embryo implantation in *A. cahirinus*.

## Methodology

### Animals

All experimental procedures in this study adhered to the Australian Code of Practice for the Care and Use of Animals for Scientific Purposes and were carried out in compliance with the ARRIVE guidelines. Spiny mice were sourced from our Monash University breeding colony and all experimental procedures were approved by the Monash Medical Centre Animal Ethics Committee (MMCB 2019/13BC).

### Tissue collection

Female spiny mice (n = 40, > 80 days and multiparous [≥ 1 litter]) were assessed at eight time points postpartum (pp): Day 0–6 and Day 10 pp. Day 10 pp animals were included to confirm embryo implantation and to rule out the possibility of embryonic diapause or delayed implantation. At all timepoints, dams were housed with the sire and actively nursed their pups. Females were anaesthetised with isoflurane prior to cardiac puncture and vaginal lavage^7^. Whole blood (0.5–1 mL) was collected in heparinised tubes, centrifuged (3000 RPM at 4 °C for 10 min) and plasma aspirated and stored at − 80 °C for later analysis. The reproductive tract with both ovaries attached was dissected out and trimmed of fat and uterine mesentery. Each ovary was then separated from its respective uterine horn and fixed in 10% buffered formalin (NBF). Uterine horns were separated by cutting through the cervix and uterine fundus and each horn fixed separately in NBF. Tissues were processed to paraffin wax, embedded and sectioned at 5 μm thick onto super-frost plus microscope slides (ThermoFisher, Australia). Slides were dried overnight and baked at 60 °C for 15 min prior to histology or immunohistochemistry.

### Hormone analysis

Spiny mouse plasmas were analysed for oestradiol-17b (E2) (ES180S-100, Calbiotech, USA), progesterone (P4) (55-PROMS-E01, ALPCO, USA). Each sample was assayed in duplicate and the validity of each assay was tested and confirmed with spike-recovery and linearity of dilution procedures following the manufacturer’s instructions.

### Ovarian and uterine morphology

Ovary and uterine sections were stained with haematoxylin and eosin as previously reported^7^. Ovaries were cut at 5um thickness and every 8^th^ section collected. Large antral follicles (> 400 μm; AF) and CLs were counted at 10X magnification using an EVOS M7000 imaging system and AFs and CLs classified as previously described in this species^11^. Endometrial thickness was measured at 20X magnification from three randomly selected fields of view (FOV) taken per animal at each time point using ImageJ software (National Institutes of Health) and mean values ± standard deviation (SD) calculated and compared across each time point. Endometrial thickness was defined as the distance from the myometrium to the uterine epithelium as described by Bellofiore, et al.^7^. As the uterine epithelium was not apparent in day 10 pp sections, endometrial thickness was not recorded at this time point.

### Immunohistochemistry

FOX01 immunostaining was used to assess luminal epithelial receptivity^15^. Tissues were dewaxed, and antigen retrieval conducted in citrate buffer pH 6.0) using a harsh retrieval method (boiled in the microwave for 9 min, simmered for 7 min and allowed to sit for 40 min). Slides were washed in Tris-buffered saline (TBS) with Tween-20 (0.05%) (TBS-T, 2X 5 min), followed by one wash in TBS. Endogenous peroxides were blocked in 3% hydrogen peroxide solution for 10 min at room temperature, prior to washing as described. Serum block (X0909, Dako, USA) was then applied for one hour at room temperature prior to primary antibody incubation (C29H4, Cell signalling, USA; 1:100) overnight at 4 °C. Negative control sections were incubated with host species IgG in place of primary antibody at the same concentration. Slides were washed thrice in TBS before incubation with secondary biotinylated antibody (BA-1000, Vectorlabs, USA; 1:250) for 30 min. Slides were then washed and incubated in avidin–biotin complex (Vectastain Elite ABC kit, Vector Labs) for 45 min at room temperature. Slides were washed once more and 3,3^′^-Diaminobenzidine (DAB) applied to each section in 30 s increments until stain developed. Slides were then rinsed in dH_2_O and counterstained in 10% Harris hematoxylin for 3–5 min. Slides were dehydrated and coverslipped as above.

### Immunofluorescence

To assess changes to vasculature and epithelium integrity postpartum, uterine tissue sections were subjected to double immunofluorescent staining for alpha smooth-muscle-actin (aSMA; #M0851, Dako) and cytokeratin (#15367, Santa-cruz, USA), as previously described^14^ with modifications. Slides were baked at 60 °C for 20 min and dewaxed through 3X changes of xylene, graded ethanols and rehydrated in dH_2_O for 5 min. Antigen retrieval was performed in citrate buffer (2.94 g Tris-sodium dihydrate in dH_2_O, pH 6.0) using a mild retrieval method (boiled for 5 min in the microwave and allowed to sit for 20 min). Slides were washed in PBS-T (0.05%) (2X 5 min), followed by one wash in PBS. Glycine solution (1.877 g of glycine in 250 mL PBS) was then applied for 30 min at 4 °C in the dark. Slides were washed thrice (as above) and incubated in 3X changes of sodium borohydride solution (1 g/100 mL dH_2_O) for 8 min and washed again. Serum block (Dako) was applied for 1 h at room temperature in the dark prior to primary antibody incubation. Mouse anti-aSMA (#M0851, Dako; 1:200) and rabbit anti-cytokeratin (#SC-15367, Santa-cruz; 1:200) were applied to each section and incubated at 4 °C overnight. Negative controls were incubated with serum block in place of primary antibodies. Slides were thrice washed in PBS prior to secondary antibody incubation. Goat anti-mouse (#A11029, Invitrogen, Australia; 488 nm) and donkey anti-rabbit (#R37115, Invitrogen; 594 nm) were applied at 1:500 to all sections in the dark for 30 min at room temperature. Slides were washed thrice in PBS and mounted with anti-fade with DAPI (#P36931; Invitrogen) prior to analysis.

### Image acquisition

Immunofluorescent images were captured using an Olympus BX43 upright fluorescent microscope and all other images captured using an EVOS M7000 imaging system. To analyse DAB stained tissues, three randomly selected FOVs were taken at 20X magnification and were blindly analysed by covering slide labels and randomly assorting slides prior to analysis using ImageJ software. For immunofluorescent tissues, three FOVs were taken of each fluorescent channel separately and blindly analysed as above. Area coverage (%) was used as a measure of immunopositive structures in the postpartum reproductive tract and each FOV was calculated to give a mean value per sample and compared against other time points. As the stratum functionalis was not apparent in day 0–2 pp uterine sections, vasculature was not assessed at these time points. Similarly, as the uterine epithelium was not apparent in day 10 pp sections, epithelial integrity (cytokeratin) and receptivity (FOX01) was not assessed at this timepoint.

### Statistical analysis

All statistical analyses were conducted using Prism 8.0 (Graphpad Prism Software Inc, USA). The Shapiro–Wilk test was used to verify normal distribution and statistical significance was set at p < 0.05 for all datasets. If data was normally distributed, all data was analysed using a one-way ANOVA and the Tukey test used for post-hoc analysis between time points and data expressed as mean ± SD.

## Results

### Comparative ovarian morphology and endocrinology between time points

**Figure 1:**
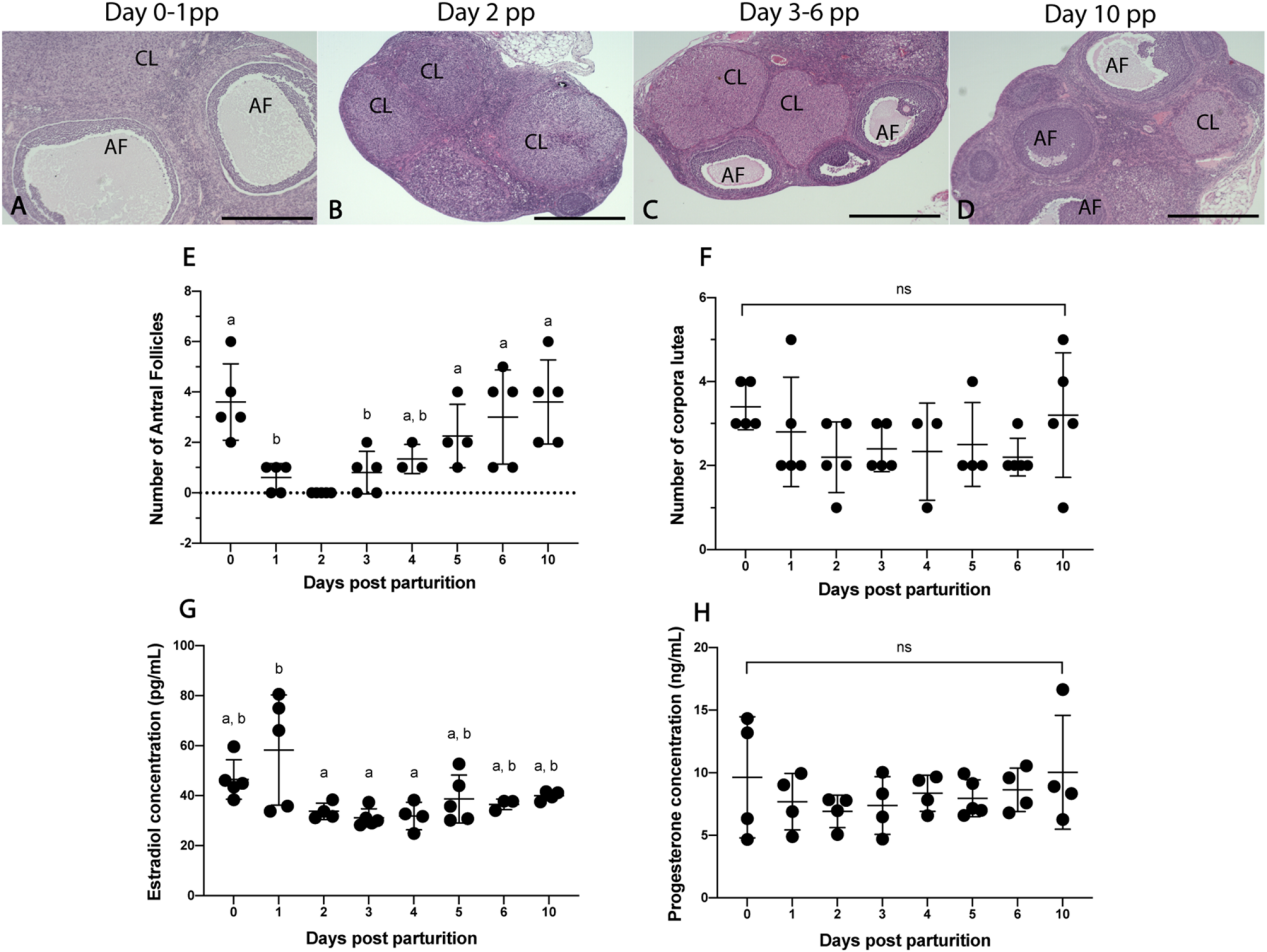
Comparative ovarian morphology and endocrinology between time points. Large antral follicles are present at all time points except for day 2 pp and corpora lutea are present at all time points (**A**–**D**). Mean antral follicle counts on days 0, 4–6 and 10 pp were similar (p > 0.05), and mean antral follicle counts on days 1, 3–4 pp were also similar (p > 0.05) (**E**). Mean number of corpora lutea was not significantly different (ns; p > 0.05) across all time points (**F**). E2 levels on days 0, 2–10 pp were similar (p > 0.05), and day 1 pp was significantly higher than days 2–4 (p < 0.05) (**G**). P4 levels were similar across all timepoints (**H**). Graphs depict mean ± SD and data with different letters (a or b) differ significantly (p < 0.05; ANOVA). Scale bars for (**A**–**D**) indicate 500um. *AF* Antral follicle, *CL* corpus luteum.

Antral follicles are present in all postpartum time points except for day 2 pp (Fig. 1). The mean number of AFs was not significantly different between days 0, 4–10 pp or between days 1–4 pp (p > 0.05; Fig. 1E). The number of CLs present in the postpartum ovary was not significantly different between any time points (p > 0.05; Fig. 1A–D,F). Circulating E2 (intra-assay CV = 9.4%) and P4 (intra-assay CV = 10.7) levels were detected in spiny mouse plasma across all time points (Fig. 1G,H). E2 levels were significantly elevated on day 1 compared to days 2–4 pp (p < 0.05; Fig. 1G), and all remaining timepoints were similar (p > 0.05). P4 concentrations were similar across all time points postpartum (p > 0.05; Fig. 1H).

### Vaginal cytology and uterine morphology between time points

**Figure 2:**
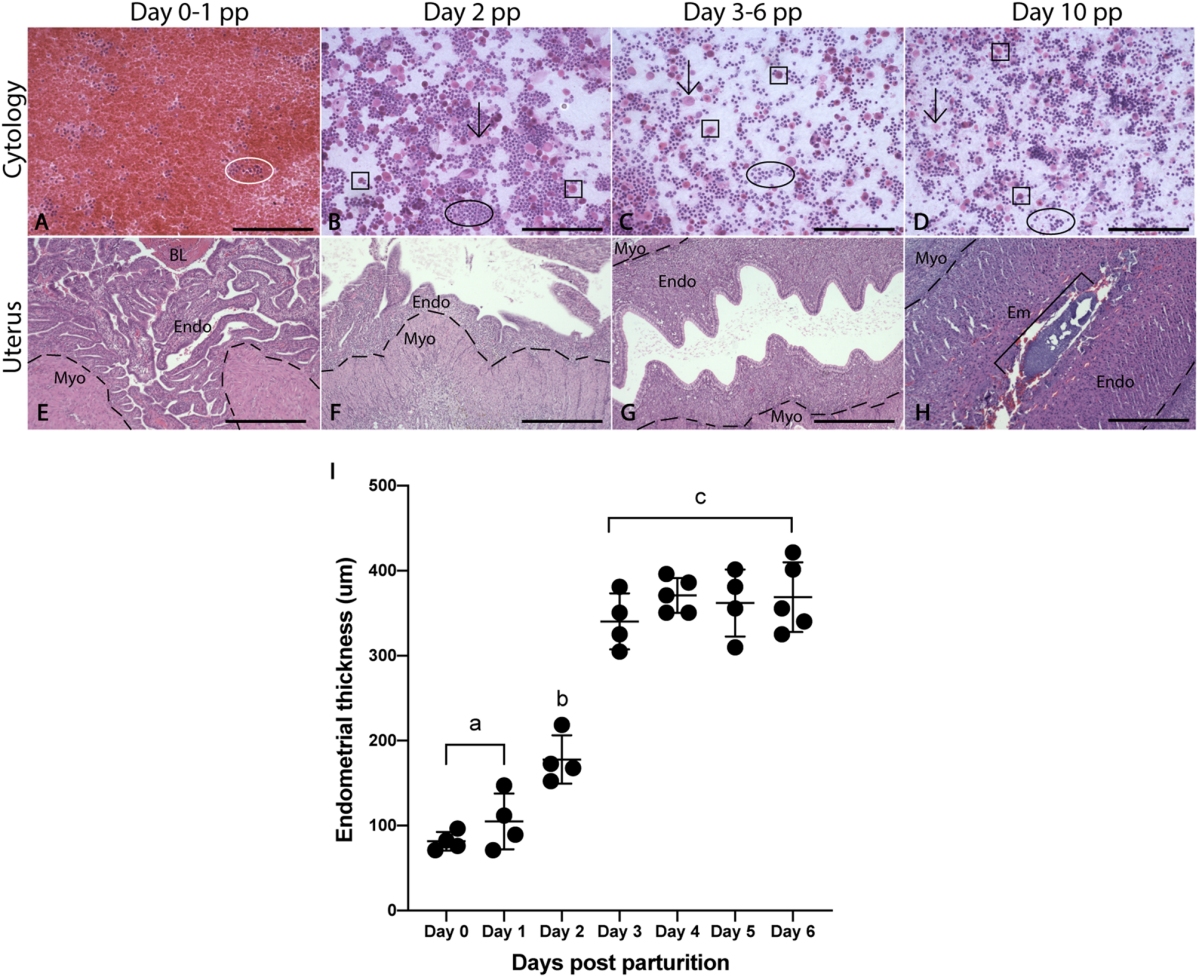
Comparative vaginal cytology and morphological changes to the postpartum uterus between time points. Vaginal cytology (**A**–**D**) across all remaining timepoints is dominated by leukocytes (circles) with a smaller number of CECs (arrows) and NECs (squares). Endometria on days 0–1 pp are thin (< 200 μm) and are not-significantly different but are significantly different to the remaining timepoints (**E**,**I**). On day 2 pp, the endometrium begins thickening rapidly and reaches maximum thickness by day 3 pp (**F**–**I**). Endometrial thickness on days 3–6 are similar (**I**). Day 10 data are excluded from analysis due to closure of the uterine lumen and regression of the luminal epithelium. Graphs depict mean ± SD and data with different letters differ significantly (p < 0.05; ANOVA). Myometrium (Myo), endometrium (Endo), embryo (Em). Scale bars are 100 μm for cytology (**A**–**D**) and 500 μm for uterine sections (**E**–**H**).

Vaginal lavages from animals on days 0–1 pp were almost entirely comprised of erythrocytes, with some samples also containing shed uterine tissue (Fig. 2A,B). By day 2 pp, erythrocytes have decreased in number and smear cytology consists of mainly leukocytes. Cytology from the remaining timepoints (days 3–6 pp, 10 pp) consisted predominantly of leukocytes and small numbers of cornified epithelial cells (CECs) and nucleated epithelial cells (NECs) (Fig. 2C,D). These cytological features were indicative of the secretory/luteal phase in spiny mice. No spermatozoa were identified in the vaginal lavages at any timepoint.

From histological evaluations, changes in the thickness of the uterine epithelium and endometrium were observed during early pregnancy in the spiny mouse uterus postpartum. The stratum functionalis was shed at parturition and the uterine endometrium remained as a very thin layer (< 300 μm) until day 3 pp (Fig. 2E–G). On days 0–1 pp, the luminal epithelium was mostly low cuboidal, but in some areas, it was absent often with remnants found sloughed into the lumen (Fig. 2E). By day 2 pp, an increase in endometrial thickness was observed with less sloughed tissue in the uterine lumen (Fig. 2F). By day 3 pp the epithelium consisted of tall, columnar cells, each with a noticeable vacuole (Fig. 2G). Moreover, the endometrium was fully grown by day 3 pp and endometrial thickness was similar to days 4–6 (Fig. 2G,I). The uterine lumen was closed by day 10 pp and the luminal epithelium had regressed (Fig. 2H–I).

### Timing and invasiveness of embryo implantation

**Figure 3:**
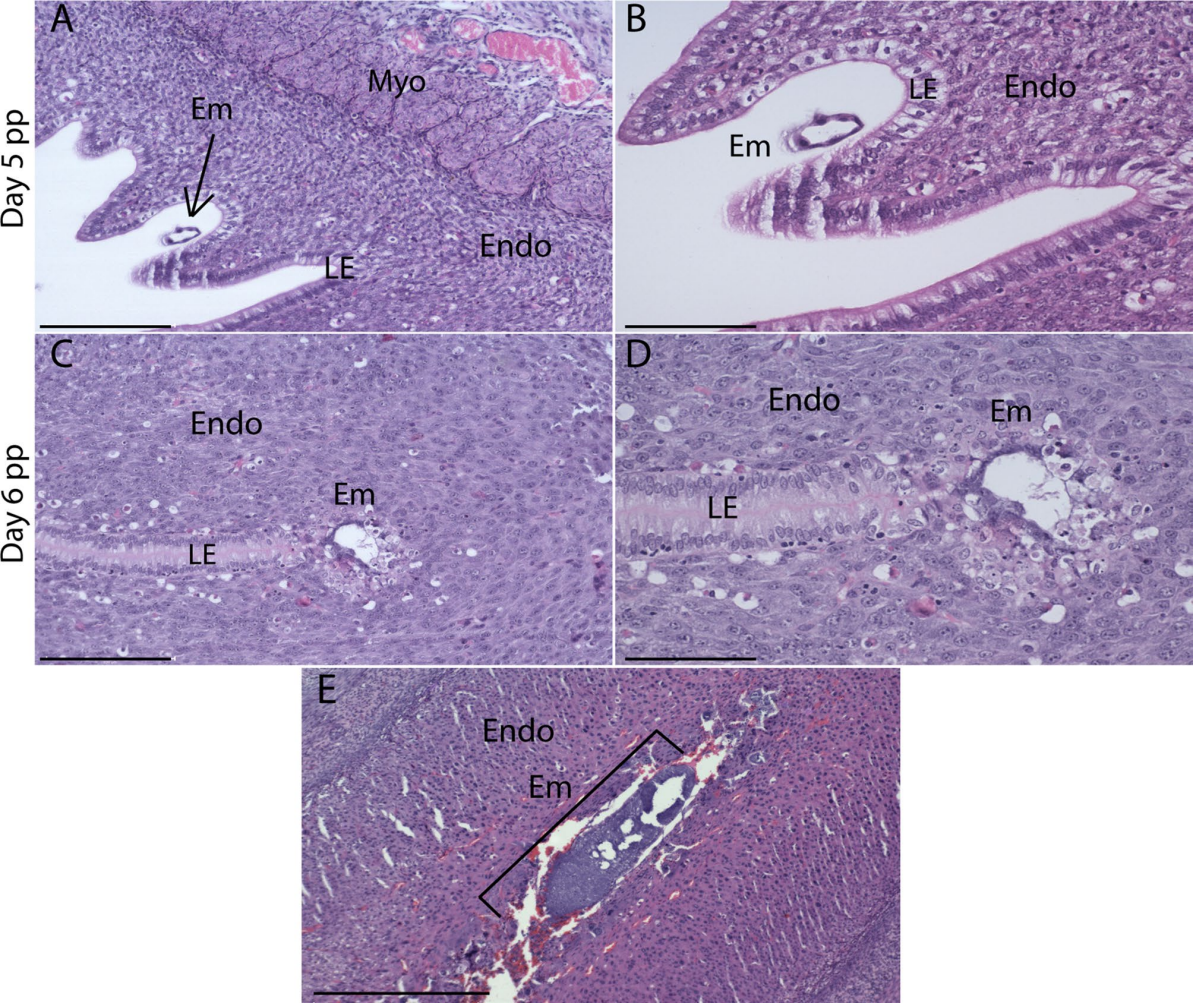
Histological sections of preimplantation embryos and implantation sites in postpartum uteri. Blastocyst (arrow) in the day 4–5 pp uterine lumen apposing sections of luminal epithelium (**A**,**B**). Embryo implantation sites were seen on day 5–6 pp (**C**,**D**). A larger, more advanced implantation site is visible in day 10 pp uteri with no remaining epithelium. Scale bars for (**A**,**C**,**E**) are 200 μm, and scale bars for (**B**,**D**) are 100 μm. Luminal epithelium (LE), embryo (Em). Endometrium (Endo), myometrium (Myo), luminal epithelium (LE), embryo (Em).

Preimplantation embryos are seen in the uterine lumen on days 4–5 pp (Fig. 3A,B) and early, superficial embryo implantation sites are evident in day 5–6 pp (Fig. 3C,D). By day 10 pp, implantation has advanced in the endometrium (Fig. 3E) as depicted by luminal closure though with minimal stromal invasion.

### Spiral arteriole remodelling and epithelial integrity

**Figure 4:**
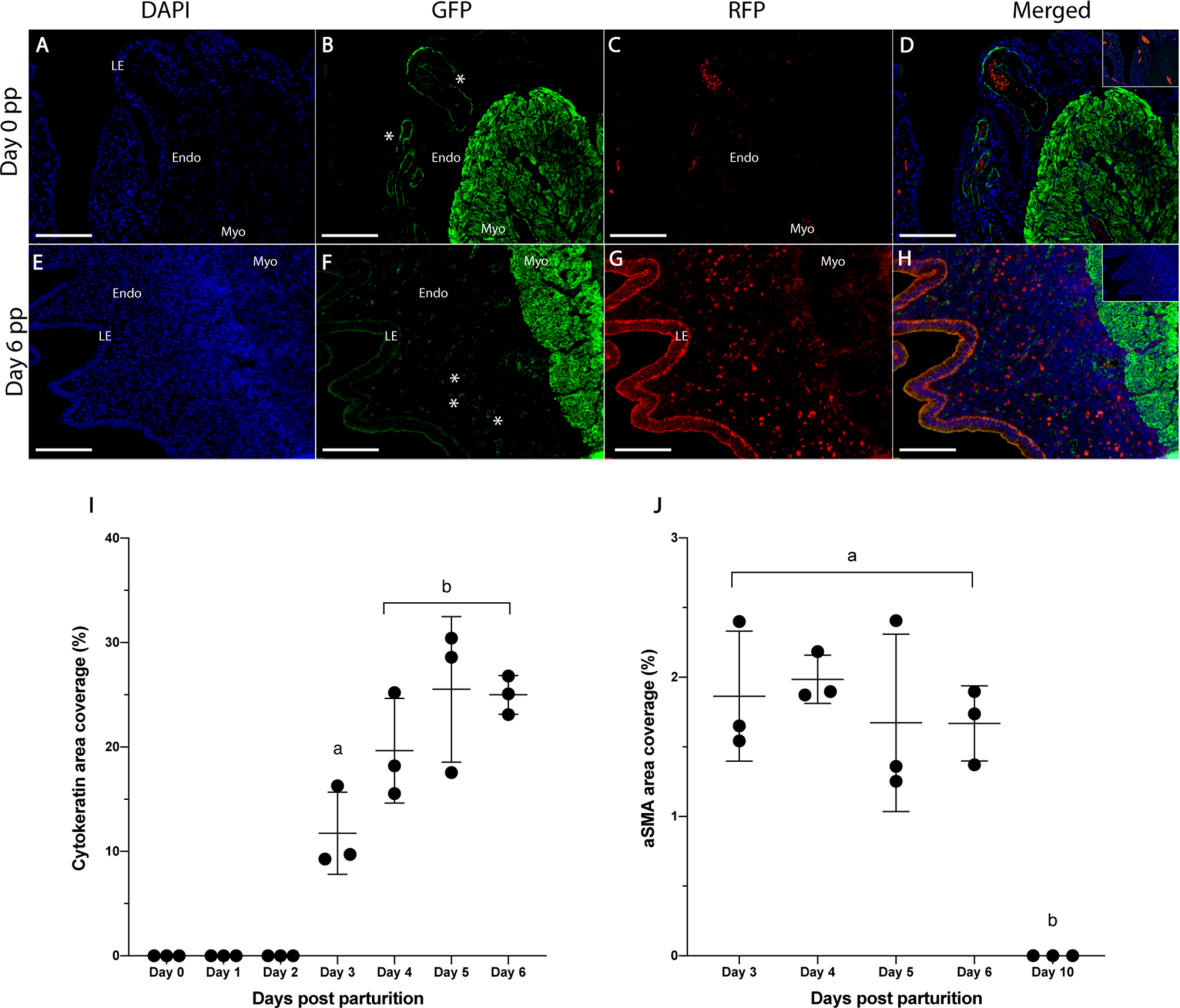
Comparative changes in cytokeratin and aSMA expression in the spiny mouse postpartum endometrium. Blue = DAPI, Green = aSMA, Red = Cytokeratin. A cluster of aSMA positive arteries in the endometrium (* in **B**) and strong aSMA positive staining in the myometrium of day 0 pp sections. Arteries in the superficial endometrium of day 6 pp sections (* in **F**) are aSMA positive. Spiral arteries were first seen on day 3 pp (**J**) and the area coverage of aSMA positive tissue was not significantly different across days 3–6 pp but was significantly higher than day 10 pp (**J**). The day 0–2 pp luminal epithelium is negative for cytokeratin (**C**), however cytokeratin is detected in the luminal epithelium by day 3 pp (**I**). Cytokeratin reached maximal expression by day 4 pp (**I**). Day 0–2 pp endometria were omitted from aSMA analysis as there was no clear stratum functionalis. As the uterine lumen was closed by day 10 pp, this timepoint was removed from cytokeratin analysis. Inset squares (**D**,**H**) are negative controls of the same tissue. Data depict mean ± SD and data with different letters differ significantly (p < 0.05; ANOVA). Scale bars for (**A**–**H**) are 100um. Endometrium (Endo), myometrium (Myo), luminal epithelium (LE).

During days 0–2 pp, large arteries were present in the basal endometrium and were immunopositive for aSMA (Fig. 4A,B,D,J); no cytokeratin expression was detected (Fig. 4C,D,I) Vasculature in the basal endometrium from day 3–6 pp tissues were smaller than at previous time points and were immunopositive for aSMA (Fig. 4E,F,H,J). Spiral artery assemblies in the superficial endometrium were immunopositive for aSMA, and the luminal epithelium was positive for cytokeratin (Fig. 4E–I). No endometrial tissues or structures were positive for aSMA on day 10 pp and cytokeratin was undetectable (Fig. 4I,J).

### Epithelial receptivity to implantation

**Figure 5:**
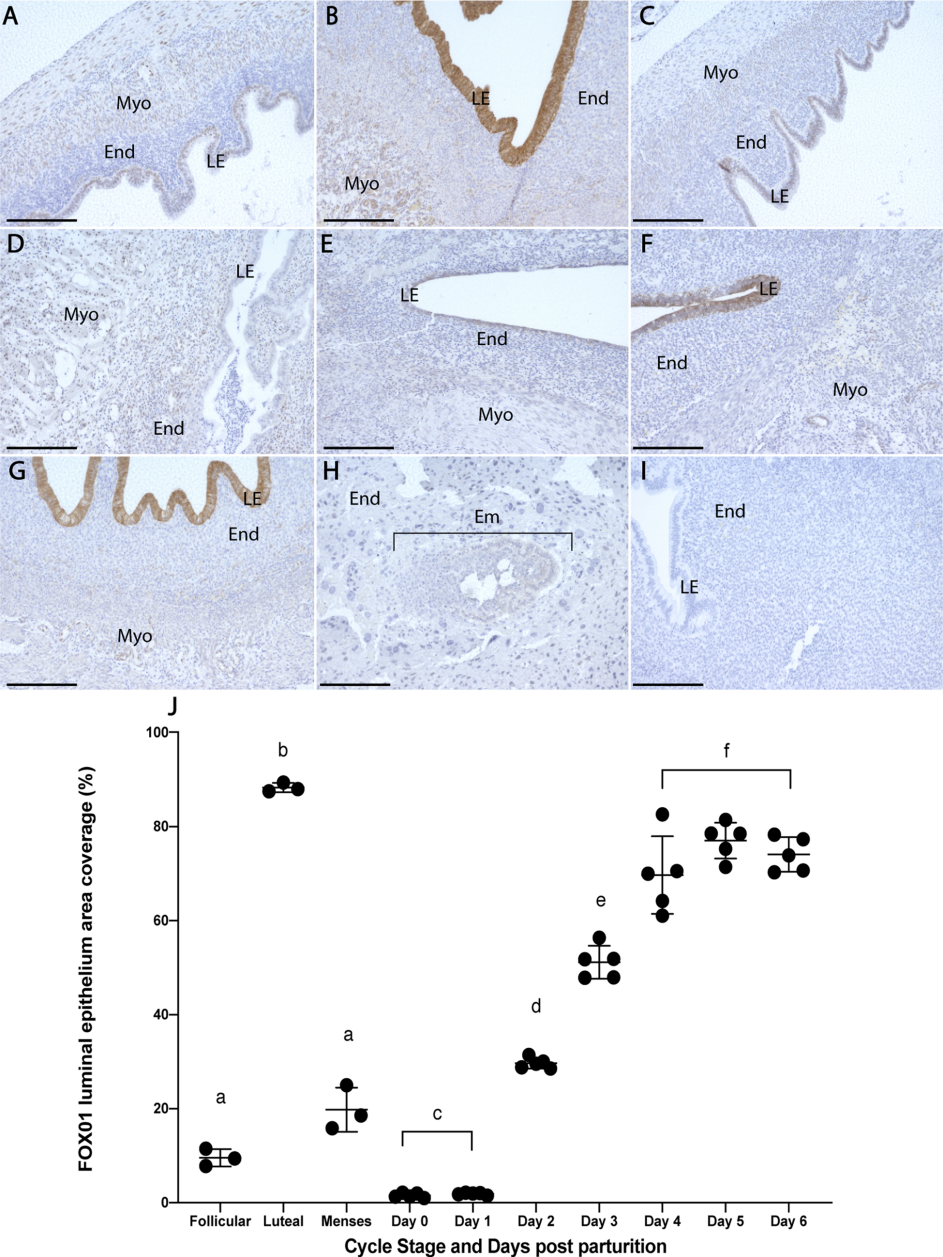
Comparative FOX01 expression during the menstrual cycle and 7 days postpartum. Follicular phase tissue (**A**) exhibited minor nuclear staining in luminal epithelial and myometrial cells. (**B**) Luteal phase luminal epithelial cells showed strong expression of FOX01 and menses phase tissue (**C**) were similar to the follicular phase. On days 0–1 pp (**D**), luminal epithelial cells were negative for FOX01, but stromal and myometrial cell nuclei were faintly positive. By day 2 pp (**E**), the luminal epithelium showed minor nuclear expression of FOX01. FOX01 expression was primarily cytoplasmic on day 3 pp (**F**) and showed minor nuclear and major cytoplasmic staining in the luminal epithelium on day 4 pp (**G**). Day 10 pp tissues (**H**) were negative for FOX01 and negative control tissue provided in (**I**). FOX01 expression was similar between follicular and menses phases but was significantly increased during the luteal phase (**J**). A significant increase of FOX01 staining was seen in the luminal epithelium on day 2 pp compared to days 0–1 pp. Similarly, day 3 pp expression was significantly higher than on day 2 pp and by day 4 FOX01 expression had peaked (**J**). Data depict mean ± SD and data with different letters differ significantly (p < 0.05; ANOVA). Scale bars indicate 200um. Endometrium (End), myometrium (Myo), luminal epithelium (LE), embryo (Em).

Minimal staining of FOX01 was detected during the follicular and menses phases; it remained restricted to the nuclei of myometrial and luminal epithelial cells (LECs; Fig. 5A,C). FOX01 expression was nuclear and cytoplasmic of LECs during the luteal phase and was significantly increased compared to the follicular and menses phases (Fig. 5B,J; p < 0.05). On days 0–1 pp, LECs were negative for FOX01, however stromal cell and myometrial cell nuclei were faintly positive (Fig. 5D). Day 2 pp LECs showed minor nuclear staining (Fig. 5E). FOX01 expression was elevated on day 3 pp with expression in the nuclei and cytoplasm of LECs (Fig. 5F) and reached maximal expression by day 4 pp (Fig. 5G,J). Day 10 pp uterine tissue, implanted embryos and negative control tissues were negative for FOX01 (Fig. 5H–I).

## Discussion

This study is the first to provide evidence for postpartum ovulation in a menstruating species. Our study conflicts with longstanding dogma of lactational amenorrhea in menstruating species^5^ and questions the regulation of the hypothalamic-pituitary–gonadal axis in *A. cahirinus.*

Spermatozoa was not observed in the vaginal lavage of females at any timepoint postpartum. However, as we are unable to confirm when mating or ejaculation took place from this study, this is unsurprising. Additionally, we are unsure of where insemination occurs within the spiny mouse female reproductive tract, which may have also limited our ability to find spermatozoa in vaginal lavages. Lastly, as spiny mice do not produce a copulatory plug^13^, further investigation of seminal deposits in the female reproductive tract would likely be invasive, and are a future direction from this research.

The absence of large preovulatory follicles on day 2 pp suggests a postpartum ovulation within 48 h of parturition for female spiny mice in our colony. Folliculogenesis resumes following PPO and large antral follicles (> 400 μm) are seen at all remaining time points. These AFs do not reach the preovulatory size seen in day 0–1 pp ovaries suggesting that they are either destined for atresia or maintained through pregnancy acting as endocrine glands until parturition. A similar pattern of follicular growth and maintenance has been reported in rodents^16,17^ and women^18^ where follicles of all sizes, except preovulatory, are seen throughout pregnancy. We cannot confirm from this study if folliculogenesis continues throughout gestation in postpartum spiny mice, but future studies of > day 10 pp tissues may reveal the presence and role of antral follicles in later stages of pregnancy.

The number of CL present in the *A. cahirinus* postpartum ovary was similar across all time points. In women, CL regresses between weeks 3–7 of gestation when the placenta assumes the role of the CL and takes over P4 secretion^19^. Contrastingly, the rodent placenta does not produce enough P4 to maintain pregnancy, therefore P4 secretion is required from corpora lutea and the placenta^20^ and there are often several CL from recent ovulations and the previous pregnancy present at parturition. Although both spiny mice and women form a haemochorial placenta^4^ at around the same point in gestation^12,21^, our results suggest a similar endocrine dependence on corpora lutea during pregnancy as in other rodent species. However, placentation in *A. cahirinus* occurs around gestational day 10–15^12^ and tissues > day 10 were not collected in this study. Moreover, as large corpora lutea were seen on day 0 pp, we cannot rule out the possibility of a pre-partum ovulation as near-term tissues were not collected in this study. Tissue collected near-term and during the period of placental development may reveal the role of the placenta and corpora lutea on progesterone secretion in *A. cahirinus*.

In mammals, a preovulatory E2 surge induces a reciprocal LH surge from the anterior pituitary, which then causes rupture of the dominant follicle, ovulation and formation of the CL^4^. To our knowledge, a PPO has not been reported in any species with a human-like menstrual cycle. The reason for this is likely to be caused by the sharp decrease in estrogen levels at birth after removal of the placenta^22^, and the onset of lactational amenorrhea^5^. However, our hormonal data suggest that an extended period of E2 stimulation up to 48hrs after parturition may exceed the ovulatory threshold in *A. cahirinus*. Interestingly, the E2 concentrations measured here are similar to late proestrus rats and postpartum mice^23,24^ suggesting this relationship may be conserved in rodents despite the pronounced differences in the reproductive cycle. However, we were unable to measure circulating LH (or FSH) levels in this study using commercially available mouse/rat or human assays. Although the amino acid sequence of peptide hormones is similar across mammalian species^25^, failure of current human and rodent assays to measure FSH and LH in the spiny mouse will now await further investigations to design species-specific antibodies for these immunoassays. Combined with our data, circulating LH levels will provide key evidence for confirming postpartum ovulation in spiny mouse dams.

Circulating progesterone levels were similar across all time points and were similar to concentrations reported during the luteal phase in women^26^ and rats^24^. However, our data differ from concentrations reported in the mouse during the luteal phase and early pregnancy^23,27^. Steroid hormones during pregnancy are known to vary significantly between menstruating species^28^ and between murine strains^23,27^. Considering this, circulating P4 levels during the luteal phase of cycling spiny mice are considerably elevated (5–15 fold) compared to postpartum females^7^. Prolactin is known to influence fertility in nursing mammals by inhibiting the release of GnRH, decreasing LH pulsatile frequency and therefore reducing ovarian steroid production^5,29,30^. As spiny mice actively nurse their young, this may explain the considerable reduction in P4 concentrations in postpartum animals compared to cycling animals.

Another explanation for the decreased P4 concentration may be changes to progesterone receptor (P4R) expression. The effects of P4 in the reproductive tract are tightly mediated by its interaction with the P4R^31^ and altered expression of the P4R has been shown to impede embryo implantation in mice^32,33^ and women^34^; generally through an impaired decidual reaction^35^. Although markers of decidualisation were not measured here, implantation sites and cellular morphology in endometria on days 5, 6 and 10 pp suggest adequate decidualisation and therefore normal P4 or P4R levels. Analysis of uterine P4R expression and concentrations of hypothalamic or pituitary hormones (including prolactin) have not been reported in this species and remain a future direction for spiny mouse research.

During the first 3 days postpartum, the endometrium is thin, consisting essentially of the stratum basalis. In menstruating species, decidualisation of the endometrium occurs solely in the stratum functionalis, which is later shed during menses or parturition^4,7^. Moreover, endometrial thickness during days 0–2 pp is similar to the endometrial thickness during the early follicular phase in this species and by day 3 pp, repair and growth of the endometrium has begun. The endometrium at this stage is structurally similar to the luteal phase in *A. cahirinus*^7^, suggesting a similar timeframe for postpartum endometrial repair and growth as during the menstrual cycle. Thus, our data show that the postpartum reproductive tract has similar structural and physiological characteristics to follicular and luteal phase reproductive tracts of spiny mice during the menstrual cycle.

Rodents undergo eccentric embryo implantation by gestational day 5 with the blastocyst forming an implantation ‘chamber’ in the superficial layer of the endometrium without fully invading the underlying stroma^15,36^. Conversely, human embryos aggressively invade the uterine luminal epithelium, and are completely embedded within the endometrial stroma (interstitial)^37^. Our observations show preimplantation embryos in the uterine lumen 4–5 days postpartum and early implantation sites in the superficial endometrium on days 5–6 pp. Larger, more advanced implantation sites were also observed in day 10 pp uteri, however minimal endometrial invasion was seen. Considering this, our data suggests that spiny mice embryos implant at a similar time postpartum and with a similar level of invasiveness to other rodents.

FOX01 has been used as a marker of luminal epithelium receptivity during the peri-implantation period in mice^15,38^, and our data demonstrates a similar expression pattern. The steady increase of epithelial FOX01 expression from day 2–4 pp is similar to the luteal phase transition in this species, and murid relatives^15,38^. Thus, it appears FOX01 also regulates epithelial receptivity to embryo implantation in spiny mouse dams, likely through modulation of the progesterone receptor. Similarly, spiral artery assemblies are seen over the same period. An advantage of the spiny mouse as a model of female reproduction is cyclical assembly of spiral arteries during the luteal phase; a feature characteristic of menstruating species^14^. Our data indicate a spatiotemporal expression pattern of endometrial aSMA during early pregnancy. Presence of aSMA positive vasculature in the endometrium by day 3 pp provides evidence for simultaneous endometrial growth/repair and spiral artery assembly; similar to the events during the menstrual cycle of this species^14^. However, immunopositive spiral arteries were not detected in the decidua on day 10 pp. In menstruating primates, invading trophoblasts break down the smooth muscle layers of spiral arteries resulting in dilated, low-resistance vessels for perfusion of the growing placenta^39,40^. Further, impaired remodelling of endometrial vasculature has been observed in women with preeclampsia and can significantly affect pregnancy outcomes^41^. It appears spiny mice experience a similar breakdown of endometrial vascular smooth muscle during early placental development (around day 10), which further validates the use of this species to investigate pregnancy/angiogenic related disorders such as pre-eclampsia. Considering this and the moderate trophoblast invasion reported here, further investigations are needed to define more clearly spiral artery function and remodelling during placental development in spiny mice.

Cytokeratins have been used as traditional markers for epithelial cell structural integrity^42^. Our data demonstrate absent cytokeratin staining in the uterine luminal epithelial cells on days 0–2 pp, which is similar to the mid-late menses phase in women^43^ and cycling spiny mice^14^ where the luminal epithelium has been destabilised and shed during menstruation. Similarly, a significant increase (p < 0.05) in immunopositive luminal epithelial cells from day 3 pp reflects the re-epithelisation and repair process occurring during the late menses phase. Structural integrity of luminal epithelial cells was apparent until day 10 pp when the luminal epithelium was largely regressed following embryo implantation; a similar process is seen during rodent embryo implantation^37^ suggesting a conserved process post-implantation in spiny mice.

While this study is the first to describe the morphology and endocrinology in postpartum spiny mice and highlights its potential as a novel laboratory model for early human pregnancy, we acknowledge several limitations to future research in this species including the lack of commercially produced suite of spiny mouse-specific antibodies. Moreover, although the spiny mouse transcriptome has been drafted^44^, a fully sequenced genome is not yet available to conduct protein sequence homology which has limited our ability to conduct a more comprehensive analysis of decidualisation and hormonal control of PPO and early pregnancy.

However, we have provided convincing, endocrine and morphological evidence for a PPO in spiny mice and described postpartum changes in angiogenesis, epithelial integrity and receptivity, and in the timing and invasiveness of embryo implantation. This study further highlights the uniqueness of *A. cahirinus* reproductive biology and its importance as a novel, small animal model of human reproduction and we encourage future research in this species to reveal more about their humanesque reproduction.

## Data Availability

The datasets generated during and/or analysed during the current study are available from the corresponding author on reasonable request.
